# Abnormal context–reward associations in an immune-mediated neurodevelopmental mouse model with relevance to schizophrenia

**DOI:** 10.1038/tp.2015.129

**Published:** 2015-09-15

**Authors:** M A Labouesse, W Langhans, U Meyer

**Affiliations:** 1 Department of Health Sciences and Technology, Physiology and Behavior Laboratory, Swiss Federal Institute of Technology (ETH) Zurich, Schwerzenbach, Switzerland; 2 Institute of Pharmacology and Toxicology, University of Zurich-Vetsuisse, Zurich, Switzerland

## Abstract

Impairments in central reward processing constitute an important aspect of the negative symptoms of schizophrenia. Despite its clinical relevance, the etiology of deficient reward processing in schizophrenia remains largely unknown. Here, we used an epidemiologically informed mouse model of schizophrenia to explore the effects of prenatal immune activation on reward-related functions. The model is based on maternal administration of the viral mimic PolyI:C and has been developed in relation to the epidemiological evidence demonstrating enhanced risk of schizophrenia and related disorders following prenatal maternal infection. We show that prenatal immune activation induces selective deficits in the expression (but not acquisition) of conditioned place preference for a natural reward (sucrose) without changing hedonic or neophobic responses to the reward. On the other hand, prenatal immune activation led to enhanced place preference for the psychostimulant drug cocaine, while it attenuated the locomotor reaction to the drug. The prenatal exposure did not alter negative reinforcement learning as assessed using a contextual fear conditioning paradigm. Our findings suggest that the nature of reward-related abnormalities following prenatal immune challenge depends on the specificity of the reward (natural reward vs drug of abuse) as well as on the valence domain (positive vs negative reinforcement learning). Moreover, our data indicate that reward abnormalities emerging in prenatally immune-challenged offspring may, at least in part, stem from an inability to retrieve previously established context–reward associations and to integrate such information for appropriate goal-directed behavior.

## Introduction

Schizophrenia is a chronic neuropsychiatric disease characterized by multiple and often co-existing psychopathological symptoms, which are typically referred to as positive, negative and cognitive symptoms.^1^ Positive symptoms include visual and/or auditory hallucinations, delusions, paranoia and major thought disorders, whereas the cognitive symptoms of schizophrenia involve disturbances in executive functions, working memory impairment and inability to sustain attention. Negative symptoms represent a class of deficits in the emotional and behavioral domains, which include asociality, poverty of speech, blunted affect and a number of distinct impairments in reward processing.^2^

Abnormalities in central reward processing are a cluster of motivational and behavioral deficits that remain largely unresponsive to currently available antipsychotic drugs and can strongly undermine the daily life quality of affected individuals.^3, 4, 5^ They include impairments in positive reinforcement learning, attribution of incentive motivation, effort-value computations and translation of reward-related information into goal-directed behavior.^6, 7^ On the other hand, the ability to enjoy pleasant stimuli seems to be largely intact in patients with schizophrenia, suggesting that schizophrenia-associated reward abnormalities emerge in the absence of anhedonia.^8^ Instead, reward-related deficits in this population may primarily stem from difficulties in using internal representations of previous rewarding experiences to drive goal-directed behaviors, which in turn would allow obtaining the desired outcomes.^6, 9, 10, 11^ These impairments seem to be restricted to positive reinforcement learning, whereas negative reinforcement or aversive learning appears to be largely intact in individuals with schizophrenia.^12, 13^

In addition to the difficulties in treating reward deficiencies, the underlying etiological mechanisms also remain largely elusive. Although antipsychotic drugs can affect reward-related functions,^9^ this class of symptoms has also been documented in unmedicated patients with schizophrenia.^14^ At least some of the reward abnormalities in schizophrenia may thus have a developmental origin and early onset.^15^ Yet, the genetic and environmental risk factors of these deficits remain largely unknown. We therefore explored whether schizophrenia-relevant abnormalities in central reward processing may be induced by prenatal exposure to infection, which has been repeatedly implicated in the etiology of schizophrenia and other neurodevelopmental disorders.^16, 17^

For this purpose, we used a well-established mouse model of maternal immune activation by the viral mimic polyriboinosinic–polyribocytidilic acid (PolyI:C).^18^ Numerous previous investigations have provided robust evidence for the emergence of long-term functional and structural brain abnormalities following prenatal exposure to PolyI:C, many of which are implicated in schizophrenia and related disorders.^18, 19^ At least some of the behavioral and cognitive abnormalities can be normalized by antipsychotic drug administration^20, 21, 22^ and emerge as a function of post-pubertal maturational processes.^21, 22, 23, 24, 25^ Hence, the prenatal PolyI:C administration model in rodents is characterized by substantial face, construct and predictive validity, and it bares epidemiological relevance to schizophrenia and related disorders. As with many other preclinical models of schizophrenia, however, the PolyI:C model has thus far only received scant attention with respect to modeling deficits relevant to the negative symptoms of schizophrenia.^26, 27^ Extending the behavioral characterization of the model to reward-related functions is thus expected to provide novel information with regard to etiological processes underlying the emergence of deficits pertinent to the negative symptoms.

Recent investigations in animal models of schizophrenia have largely focused on incentive motivation abnormalities that associate with reduced exertion of effort.^28, 29^ Because patients with schizophrenia exhibit impairments in other distinct neuropsychological processes that are independent of effort-related behavior, such as for example positive reinforcement learning or reward-based decision making,^9, 10, 11, 12^ we focused our attention on Pavlovian-based tests that require only minimal levels of effort exertion. We explored the effects of prenatal PolyI:C-induced immune challenge on Pavlovian-based reward behaviors using a well-established contextual reward conditioning task, namely the conditioned place preference (CPP) test.^30^ Performance in the CPP test is dependent on various hippocampal and cortico-striatal circuits,^31^ which are known to be altered in schizophrenia.^31, 32, 33, 34, 35^ Using the CPP test, we evaluated context–reward associations using a natural reward (sucrose) and the psychostimulant drug cocaine. We also assessed the effects of prenatal immune activation on hedonic and neophobic responses to natural sucrose reward in attempts to identify possible relationships between positive reinforcement learning and anhedonic and/or neophobic behavior. Finally, we used a contextual fear conditioning paradigm to explore the effects of prenatal immune activation on negative reinforcement learning that involves contextual processing of aversive events.

## Materials and methods

### Maternal immune activation during pregnancy

C57BL6/N pregnant dams on gestation day 17 were randomly assigned to receiving either PolyI:C (potassium salt; Sigma-Aldrich, Buchs, Switzerland) or vehicle (NaCl). Adult offspring born to immune-challenged and control mothers were included in the study. All procedures had been previously approved by the Cantonal Veterinarian's Office of Zurich and are fully described in the Supplementary Methods.

### Behavioral characterization of offspring born to immune-challenged and control mothers

We evaluated positive contextual conditioning for sucrose and cocaine using standard CPP paradigms.^30, 36^ In addition, we evaluated whether anhedonic behavior and anxiety-related sucrose neophobia could represent confounders influencing the behavioral responses in the sucrose CPP. Finally, to explore the effects of prenatal immune activation on negative contextual conditioning, we measured context–fear-associative learning using a standard contextual fear conditioning paradigm.^37^ The behavioral paradigms are fully described in the Supplementary Methods.

### Statistical analyses

All data met the assumptions of normal distribution and equality of variance, or were transformed accordingly. All data were analyzed using Student's *t-*tests (two tailed), parametric analysis of variance or analysis of covariance, followed by Fisher's least significant difference *post hoc* group comparisons, or non-parametric Mann–Whitney tests. Statistical analyses are fully described in the Supplementary Methods.

## Results

### Prenatal immune activation reduces contextual conditioning for sucrose reward

**Figure 1:**
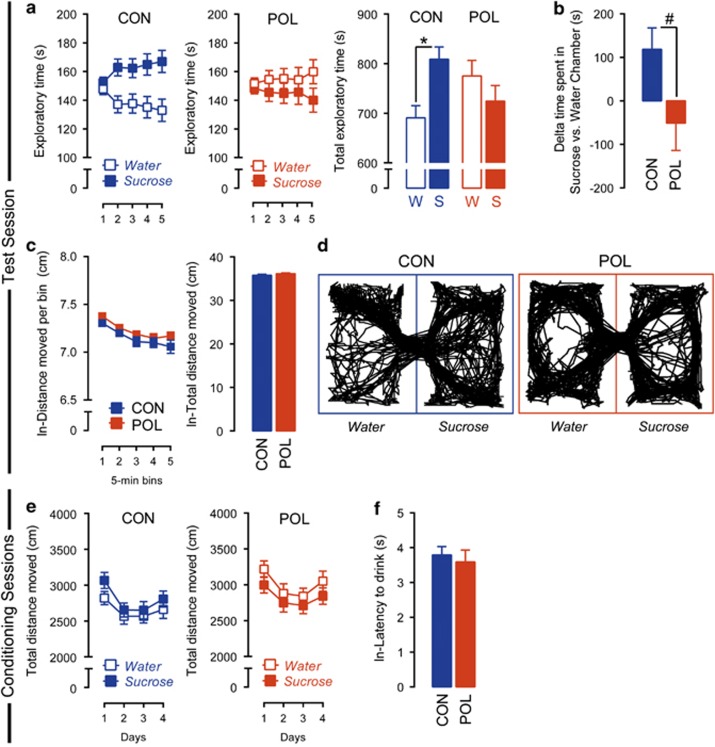
Prenatal immune activation impaired context–reward associations elicited by natural sucrose reward. (**a**) Reduced place preference for sucrose in offspring born to PolyI:C-exposed (POL) mothers as compared with control (CON) mothers. The line plots show exploratory time in the water-paired (W) and sucrose-paired (S) chambers as a function of 5-min bins during the test phase, and the bar plots show the total exploratory time in the two chambers across the entire test period. *P<0.05, based on *post hoc* comparisons. (**b**) Reduced place preference for sucrose in POL offspring as assessed by the relative difference in the time spent in the sucrose- vs water-paired chamber. ^#^*P*<0.05, based on a Mann–Whitney test. (**c**) Intact locomotor activity in POL and CON offspring during the place preference test phase. The line plots show the log-transformed distance moved in both chambers as a function of 5-min bins during the test phase, and the bar plots show the distance moved across the entire test period. (**d**) Computer-generated exploration patterns for representative POL and CON offspring in the two (water- or sucrose-paired) chambers during the test phase. (**e**) Intact locomotor activity in POL and CON offspring during the conditioning phase of the test. The line plots show the total distance moved in the water- and sucrose-paired chambers on conditioning days 1–4. (**f**) Intact log-transformed latency to consume sucrose in the sucrose-paired chamber on the last conditioning day in POL and CON offspring. *N*_(CON)_=35, *N*_(POL)_=35. All values are mean±s.e.m.

In a first series of investigations, we explored the effects of prenatal immune activation on contextual appetitive conditioning for sucrose using a standard sucrose CPP test. Following conditioning, the exploration time between the sucrose-paired and the water-paired (control) chamber was used to assess contextual appetitive conditioning in adult control and PolyI:C offspring. The exploration time was recorded during a 25-min test period and analyzed as a function of 5-min bins. As shown in Figure 1a, control offspring displayed a clear preference toward the sucrose-paired chamber, indicating intact contextual appetitive conditioning. This preference was fully abolished in PolyI:C offspring, so that they persistently explored both chambers to a similar extent during the entire test session (Figure 1a). Statistical support for these observations was obtained by the significant interaction between chamber and prenatal treatment (*F*_(1,68)_=4.51, *P*<0.05) and by the subsequent restricted analyses of variance revealing a significant difference between the sucrose-paired and the water-paired chambers in control (*F*_(1,34)_=5.78, *P*<0.05), but not in PolyI:C offspring (Figure 1b). Similar results were obtained when sucrose CPP was indexed by the relative difference in the time spent in the sucrose- vs water-paired chamber (Figure 1b). These analyses revealed that control offspring spent ~120 s more in the sucrose-paired chamber than in the water-paired chamber, whereas PolyI:C offspring spent ~40 s less in the sucrose chamber relative to the water chamber (Figure 1b). This led to a significant group difference (Mann–Whitney test: *Z*=−2.091, *P*<0.05) in the relative time spent in the sucrose- vs water-paired chamber. Representative exploration patterns of PolyI:C and control offspring can be found in Figure 1d.

Notably, the effects of prenatal immune activation on sucrose CPP emerged in the absence of any difference in general locomotor activity during the test session (Figure 1c) or during the conditioning sessions (Figure 1e). These effects also emerged independently of any differences in general food or fluid intake measured in the same cohort of animals (data not shown), supporting the notion that differences in hunger or thirst did not affect the behavioral readouts of interest. We also assessed the strength of appetitive conditioning by measuring the latencies to consume the sucrose solution on the last conditioning day. This analysis revealed highly similar latencies in control and PolyI:C offspring, with the majority (~90%) of animals starting to consume the sucrose reward within the first 80 s (see Figure 1f showing the log-transformed latency to drink on the last conditioning day). None of the animals consumed water in the water-paired chamber within the first 5 min of the last conditioning session (data not shown). Together these results suggest that prenatal immune activation leads to abnormalities in contextual appetitive conditioning for natural sucrose reward in the absence of possible confounding effects on locomotor activity and/or deficits in appetitive conditioning *per se*.

### Prenatal immune activation does not affect hedonic or neophobic responses to sucrose

**Figure 2:**
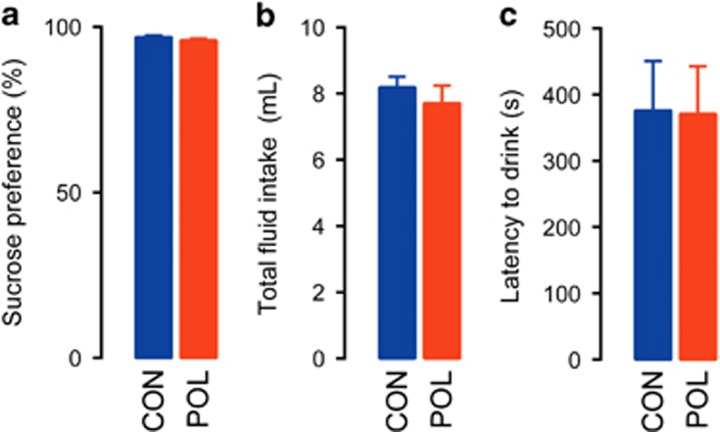
Prenatal immune activation did not affect sucrose preference or sucrose neophobia. (**a**) Intact sucrose preference in offspring born to PolyI:C-exposed (POL) or control (CON) mothers as indexed by the percentage score [sucrose consumption/(total liquid consumption) × 100%] obtained during a 24-h test period using a 30% sucrose solution; *N*_(CON)_=8, *N*_(POL)_=8. (**b**) Intact total fluid (water+sucrose solution) consumption during the 24-h sucrose preference test period. (**c**) Intact sucrose neophobia in POL and CON offspring as assessed by the latency to start consuming sucrose solution (30% sucrose) upon its first presentation. *N*_(CON)_=8, *N*_(POL)_=8. All values are mean±s.e.m.

Next, we evaluated whether the PolyI:C-induced deficits in CPP for sucrose may be associated with anhedonic behavior toward the primary reinforcer, that is, an inability to experience pleasure from stimuli that others normally find pleasurable. To this end, we compared control and PolyI:C animals in a two-bottle sucrose preference test, which is frequently used to assess anhedonic behavior.^38^ We used the same 30% sucrose solution as in the preceding CPP test so as to provide a direct comparison between the two experimental paradigms. As shown in Figure 2a, PolyI:C and control offspring similarly displayed a marked (~95%) preference toward the sucrose solution, indicating intact hedonic reactions to the reinforcer in both prenatal treatment groups. Likewise, total fluid (water and sucrose solution) intake was also highly comparable between PolyI:C and control offspring during the entire 24-h test (Figure 2b).

We further explored whether PolyI:C offspring might show altered neophobic responses to the same 30% sucrose solution. In this test, sucrose neophobia was indexed by the latency to start consuming sucrose solution when presented the first time in a familiar context. These analyses revealed no significant group differences (Figure 2c), indicating intact neophobic reaction to the sucrose reinforcer in both prenatal treatment groups.

### Prenatal immune activation does not affect aversive contextual conditioning

Given that prenatal immune challenge robustly affected the expression of positive contextual conditioning (Figure 1), we next sought to explore whether deficits in contextual learning would extend to the negative valence domain. We thus subjected PolyI:C and control offspring to a contextual fear conditioning task, whereby the acquisition and expression of contextual fear was indexed by the percent time freezing.

**Figure 3:**
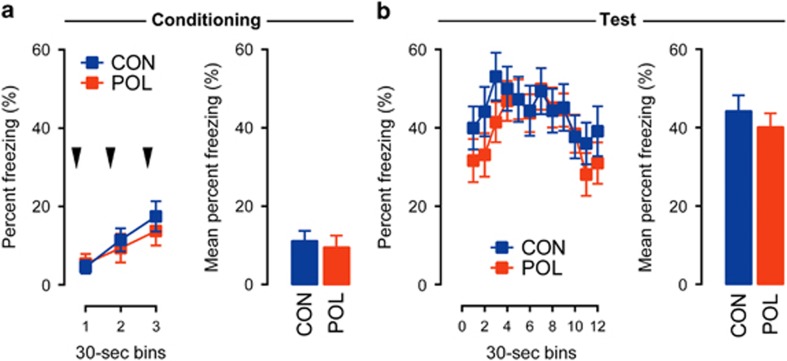
Prenatal immune activation did not affect the acquisition and expression of contextual fear conditioning. (**a**) Intact acquisition of fear conditioning in offspring born to PolyI:C-exposed (POL) or control (CON) mothers. The line plots show percent time freezing during the 30-s post-shock bins on the day of conditioning, and the bar plots show the mean percent time freezing across the entire post-shock period. The black arrowheads indicate the occurrence of the foot-shock stimulus. (**b**) Intact expression of contextual fear memory in POL and CON offspring. The line plots display percent time freezing as a function of 30-s bins during the contextual memory test phase, and the bar plots show the mean percent time freezing across the entire 6-min test period. *N*_(CON)_=16, *N*_(POL)_=16. All values are mean±s.e.m.

During the conditioning phase of the contextual fear test, the amount of percent time freezing generally increased across the three successive post-shock periods (Figure 3a), indicating that the animals developed a noticeable fear response as a result of shock exposure. The development of such fear responses was highly comparable between the experimental groups (Figure 3a). Hence, prenatal immune activation did not significantly affect the acquisition of contextual fear. Contextual fear memory was then evaluated 24 h following conditioning and was indexed by the expression of conditioned fear toward the context, in which aversive conditioning took place. Again, PolyI:C and control offspring displayed comparable levels of conditioned fear toward the context (Figure 3b), suggesting that prenatal immune activation did not affect the retention of previously acquired contextual fear memories. These data demonstrate that prenatal immune activation does not alter contextual learning belonging to the negative valence domain, despite the fact that it impaired positive contextual conditioning for sucrose reward (Figure 1).

### Prenatal immune activation enhances contextual conditioning for cocaine reward

**Figure 4:**
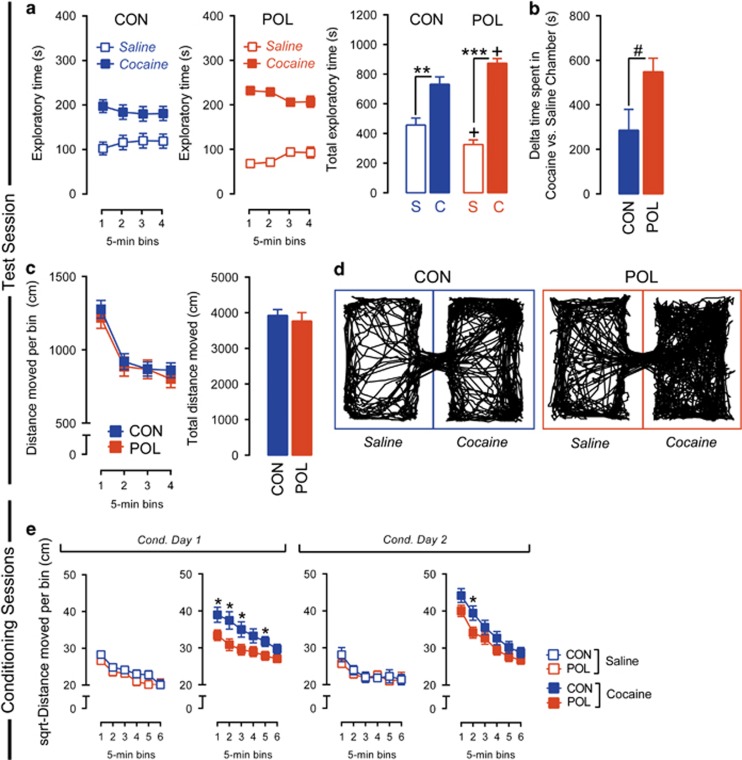
Prenatal immune activation enhanced context–reward associations elicited by cocaine. (**a**) Enhanced place preference for cocaine in offspring born to PolyI:C-exposed (POL) as compared with control (CON) mothers. The line plots show exploratory time in the saline-paired (S) and cocaine-paired (C) chamber as a function of 5-min bins during the test phase, and the bar plots show the total exploratory time in the two chambers across the entire test period. ^**^P<0.01 and ^***^P<0.001, based on *post hoc* comparisons in CON and POL offspring, respectively. ^+^*P*<0.05, signifying a significant difference in the total exploratory time of the cocaine, and saline chambers, respectively, in POL as compared with CON offspring; based on *post hoc* comparisons. (**b**) Enhanced place preference for cocaine in POL offspring as assessed by the relative difference in the time spent in the cocaine- vs saline-paired chamber. ^#^*P*<0.05, based on a one-way analysis of variance. (**c**) Intact locomotor activity in POL and CON offspring during the place preference test phase. The line plots show the distance moved in both chambers as a function of 5-min bins during the test phase, and the bar plots show the total distance moved in the two chambers. (**d**) Computer-generated exploration patterns for representative POL and CON offspring in the two (saline- or cocaine-paired) chambers during the test phase. (**e**) Reduced cocaine-induced locomotor responses in POL as compared with CON offspring during the conditioning phase of the test. The line plots show the square-root-transformed distance moved as a function of 5-min bins in the saline- and cocaine-paired chambers on conditioning days 1 and 2. *P<0.05, reflecting a significant reduction in the locomotor response to cocaine in POL as compared with CON offspring at individual time points. *N*_(CON)_=18, *N*_(POL)_=18. All values are mean±s.e.m.

Finally, we explored whether prenatal immune activation would alter contextual reward learning under conditions in which the reward takes the form of the psychostimulant drug cocaine. To this end, PolyI:C and control offspring were subjected to a CPP for cocaine, and the exploration time between the cocaine- and saline-paired chambers was used to index contextual conditioning for cocaine. As expected,^36^ conditioning led to a robust preference for the cocaine-paired chamber in both prenatal treatment groups, whereby the time spent in the cocaine-paired chamber was significantly higher than the time spent in the saline-paired chamber (main effect of drug: *F*_(1,34)_=55.29, *P*<0.001) (Figure 4a). Interestingly, prenatal immune activation potentiated this effect, so that the exploration time in the cocaine-paired chamber was longer in PolyI:C offspring compared with controls. This was reflected by the significant interaction between chamber and prenatal treatment (*F*_(1,34)_=5.48, *P*<0.05) and by the significant difference in the exploration time of cocaine- and saline-paired chambers between the two prenatal treatment groups (*F*_(1,34)_=5.48, *P*<0.05) revealed in subsequent restricted analyses of variance (Figure 4a). Similar results were obtained when cocaine CPP was indexed by the relative difference in the time spent in the cocaine- vs saline-paired chamber (Figure 4b): although control offspring spent ~300 s more in the cocaine-paired chamber than in the saline-paired chamber, PolyI:C offspring spent twice the amount of time (~600 s) in the cocaine-paired chamber as compared with the saline-paired chamber, leading to a significant prenatal group difference (*F*_(1,34)_=5.48, *P*<0.05). These analyses remained significant after inclusion of locomotor activity as covariates in an analysis of covariance (day 1 included as a covariate: *F*_(2,33)_=4.63, *P*<0.01; day 2 included as a covariate: *F*_(2,33)_=4.21, *P*<0.01). Prenatal treatment did not affect general locomotor activity, as indexed by the total distance moved on the test day (Figure 4c). Representative exploration patterns of PolyI:C and control offspring can be found in Figure 4d.

Aberrant expression of cocaine-associated place preference could emerge as a consequence of altered cocaine sensitivity. To explore this possibility, we analyzed the locomotor responses to cocaine administration during the conditioning sessions. As expected, cocaine administration increased locomotor activity compared with saline treatment (main effect of drug (*F*_(1,34)_=81.38, *P*<0.001) in square-root-transformed data). Subsequent *a priori* separation of cocaine and saline data revealed that the cocaine-induced enhancement of locomotor activity was apparent in both experimental groups. This locomotor-stimulating effect of cocaine was, however, blunted in PolyI:C offspring compared with controls, especially within the first 15 min after drug injection (Figure 4e). These observations were supported by the presence of a significant main effect of prenatal treatment (*F*_(1,34)_=4.33, *P*<0.05) and by a significant interaction between prenatal treatment and bins (*F*_(5,70)_=2.50, *P*<0.05). Subsequent *post hoc* comparisons revealed significant group differences at several individual time points after cocaine treatment (all *P*<0.05). Significant group differences in locomotor activity were only observed following cocaine, but not saline, administration (Figure 4e). An additional analysis of the mean meanders (that is, degrees of turn angle divided by distance moved) was performed to assess behavioral patterning as an index of behavioral stereotypy. These analyses revealed no group differences, suggesting that the PolyI:C-induced differences in cocaine-induced locomotor activity are not driven by altered locomotor patterning (Supplementary Figure S1).

## Discussion

The present study demonstrates that prenatal exposure to viral-like immune activation alters reward processing in contextual preference tasks. Offspring of immune-challenged mothers exhibited deficits in the expression of sucrose CPP, whereas they did not display alterations in the sucrose preference or neophobia tests. These data thus suggest that prenatal immune activation impairs the ability to adequately engage context–reward associations for natural reinforcers such as sucrose without changing hedonic or neophobic responses to the reinforcer itself. Moreover, the deficits in sucrose CPP were not associated with any changes in locomotor activity, so that the reward-related phenotype of immune-challenged offspring is not secondary to aphasia or fatigue. We also did not detect any differences in general food or fluid intake, suggesting that the effects demonstrated herein are not secondary to differences in internal states such as hunger or thirst. Rather, the present data suggest that prenatal immune activation leads to abnormalities in the ability to translate previously established context–reward associations into adequate goal-directed exploratory behavior. In support of this notion, both experimental treatment groups similarly displayed short latencies to consume the sucrose solution on the last conditioning day, indicating that the ability to create context–reward associations appears to be intact in immune-challenged offspring. Our data thus suggest that the PolyI:C-induced deficits in sucrose CPP cannot be attributed to primary defects in reinforcement learning and/or incentive salience attribution, but may rather be precipitated by an inability to retrieve previously established context–reward associations and to integrate such information for appropriate goal-directed behavior.

A number of neuropsychological and cognitive processes could underlie these effects. For example, they may involve impairments in the capacity to reintegrate previously acquired information about contexts (that is, two segregated chambers during conditioning) into a novel contextual entity (one large chamber made of two subchambers during the test day), a phenomena known to rely on prefrontal–hippocampal functions.^31^ These behavioral deficits may also depend on the capacity to integrate information about the value of actions and rewards, largely dependent on orbitofrontal and striatal activity.^39, 40, 41^ The place preference deficits could also emerge as a result of altered decision making when facing two competing choices, or as a result of a reduced capacity for uncertainty-driven exploration. The latter is characterized by the ability to generate exploratory behaviors in environments where reward outcomes are uncertain. Decision-making and uncertainty-driven exploration are both largely dependent on the integrity of the prefrontal cortex.^12, 42^ Finally, the reward deficits described herein could be induced by abnormal cortico-striatal functions that translate decisions into actions, a process that is further modulated by contextually relevant hippocampal signals.^31^ One limitation of our study is that we did not attempt to identify the neuronal substrates of altered reward processing following prenatal immune challenge. Several previous investigations, however, have repeatedly demonstrated a number of cellular and neurochemical abnormalities in reward-relevant brain areas of immune-challenged offspring, including reduced levels of dopamine and impaired expression of GABAergic markers in hippocampal and prefrontal tissues^24, 26, 43, 44, 45^ and deficient dopamine receptor functions in prefrontal and striatal regions.^25, 46, 47, 48^ Future studies will be necessary to determine whether neural abnormalities in these mesocorticolimbic and hippocampal circuits may functionally contribute to the reward-related deficits emerging following prenatal immune challenge. It will also be interesting to determine whether the effects of prenatal immune activation extend to other neuropsychological impairments of the reward domain such as, for example, incentive motivation and exertion of effort.

Another main finding of the present study is that prenatal immune activation also altered contextual learning for the psychostimulant drug cocaine. Unlike in the natural reward (sucrose) condition, however, offspring of immune-challenged mothers showed enhanced place preference for the drug reward. At the same time, immune-challenged offspring displayed reduced cocaine-induced locomotor hyperactivity compared with cocaine-treated control offspring. The analyses of locomotor patterning further suggested that these effects represent genuine effects on locomotor activity rather than possible differences in stereotyped behavior. Moreover, the PolyI:C-induced effects on cocaine CPP scores remained significant after controlling for differences in locomotor activity. Our findings thus reveal an intriguing dissociation between the locomotor-enhancing effects of cocaine and its reward-related properties, whereby reduced locomotor activity in prenatally challenged offspring are concomitant to an enhancement of context–cocaine associations. Our data are in line with the notion that the locomotor-stimulating and reward-related effects of cocaine partly rely on distinct neuronal mechanisms and brain subregions.^49, 50^ Accordingly, the attenuation of cocaine-induced hyperactivity may primarily involve reduced dopamine transporter availability in the core region of the nucleus accumbens, which mediates the immediate locomotor effects of acute cocaine treatment.^51, 52, 53^ Conversely, context–cocaine reward behaviors critically depend on integrated circuits within hippocampal and cortico-striatal loops engaging the nucleus accumbens shell, which together promote context–drug learning, attribution of incentive and predictive properties to contexts, and retrieval of memories.^49, 50, 54, 55^ These processes emerge after repeated context–cocaine pairings over several days, which likely involves synaptic remodeling and plasticity-related changes within mesocorticolimbic regions.^56, 57^ For example, an elegant study by Muñoz-Cuevas *et al.*^56^ identified rapid spine gain in prefrontal pyramidal neurons following cocaine administration, demonstrating that persistent new spines correlate with the magnitude of cocaine place preference, but not with locomotor sensitization to the drug. Enhanced neuroadaptive changes in mesocorticolimbic structures, together with an overlearning of drug-related cues,^55^ could thus provide a plausible explanation for the increased cocaine place preference emerging in PolyI:C offspring. Even though indirect evidence for this possibility exists based on previous neurochemical and neuroanatomical studies,^25, 46, 47, 48^ future investigations will be required to delineate the exact neural mechanisms implicated in these effects.

It also remains elusive whether the diametrically opposite patterns of reward abnormalities (reduced and enhanced reward responses to sucrose and cocaine, respectively) may arise from defects in the same neural circuits, or rather whether they represent independent pathological entities. According to some theoretical accounts, however, seemingly contradictory reward-related phenotypes, such as reduced place preference for sucrose and enhanced place preference for cocaine, could indeed be induced by mutual neural mechanisms. One of these mechanisms may relate to abnormal tonic vs phasic dopamine signaling: it has been hypothesized that abnormalities in tonic vs phasic dopaminergic signals could result in deficient signal-to-noise detectability.^58^ According to this theory, sucrose stimuli would fail to elicit a sufficiently large phasic dopamine response to be detected above ‘noise' thresholds in immune-challenged offspring,^58, 59, 60^ whereas cocaine may elicit stronger phasic signals in immune-challenged offspring compared with controls.^59, 60^ The latter may occur because of the existence of abnormally large phasic vs tonic dopamine responses to strong dopaminergic stimulants such as cocaine (or cocaine-related cues), similarly to what has been proposed in the context of the aberrant salience hypothesis of schizophrenia.^11, 61, 62^

An alternative (but not mutually exclusive) explanation for the differential effects of prenatal PolyI:C exposure on sucrose- and cocaine-induced CPP relates to a recent observation suggesting that exposure to natural and drug rewards induce partly distinct neuronal changes, in particular within the prefrontal-to-accumbens glutamate pathway and/or downstream medium spiny neurons.^63, 64^ For example, rat studies have shown that cocaine exposure, but not food exposure, elicits prefrontal-dependent increases in spine size and strength in accumbal medium spiny neurons.^65^ In addition, a recent study has identified differential expression of the epigenetic regulator MeCP2 and some of its target genes (PP1Cβ or reelin) following passive- or self-administration of cocaine vs food.^66^ In view of our findings, we speculate that neurodevelopmental disruption by prenatal PolyI:C exposure may differentially alter some of the key neuronal substrates underlying the rewarding effects of natural (for example, sucrose) and drug (for example, cocaine) rewards, which in turn may critically determine the direction of reward abnormalities depending on the rewards' nature.

Whatever precise mechanisms involved, our results are consistent with and corroborate some of the reward-related dysfunctions present in schizophrenia and other neurodevelopmental animal models relevant to this disorder. In line with our observations in the sucrose preference test of anhedonia, patients with schizophrenia typically retain the ability to enjoy pleasurable stimuli.^6, 42^ Furthermore, our results match the large body of evidence suggesting that individuals with schizophrenia show intact negative reinforcement learning, similarly to what we have observed in the contextual fear conditioning test. On the other hand, patients with schizophrenia display clear deficits in positively motivated learning tasks involving rewarding stimuli.^67, 68^ Besides other abnormalities, affected individuals seem to be incapable of translating value/effort-related information into goal-directed action.^6^ They also show difficulties in their ability for uncertainty-driven exploration.^12, 42^ Both of these deficits seem to critically involve prefrontal dysfunctions, which we believe might be implicated in the sucrose place preference deficits that emerged following prenatal immune activation.

It is also interesting to note that several studies have reported a high prevalence of substance use disorder in schizophrenia, including abuse of psychostimulant drugs such as cocaine.^69, 70^ The findings from our preclinical model suggests that maternal infection could represent one of the neurodevelopmental factors triggering the appearance of an addiction-prone phenotype in patients with schizophrenia, although epidemiological data supporting such an association are missing thus far. Yet, our findings are in line with the previous work in other schizophrenia-relevant animal models documenting altered responses to natural or drug rewards, including models that are based on neonatal ventral hippocampal lesion,^71, 72, 73^ prenatal infection^74, 75^ or mutant expression of DISC1.^76^ Altogether, these observations provide support for the theory that the predilection for addictive behavior would be ingrained in schizophrenia neuropathology.^77^ Indeed, it has been proposed that the neuropathological abnormalities that emerge in the brain of individuals with schizophrenia across neurodevelopment create a state of ‘endogenous sensitization', so that functional hyper-responsiveness of dopamine-related circuits, coupled with reduced inhibitory control arising from cortical structures (some of the hallmark features of schizophrenia), could result in a hypersensitivity to the rewarding effects of drugs.^77, 78^ Although current knowledge does not readily allow confirming or refuting this hypothesis, our findings support the notion that neurodevelopmental changes in mesocorticolimbic circuits, which emerge in response to prenatal immune challenge, could potentially also be responsible for drug-related behavioral abnormalities.

As extensively reviewed elsewhere,^18, 19^ the prenatal PolyI:C administration model in rodents is characterized by substantial face, construct and predictive validity, and it bares epidemiological relevance to schizophrenia and related disorders. As with many other preclinical models of schizophrenia, however, the PolyI:C model has thus far only received scant attention with respect to modeling aspects relevant to the negative symptoms of schizophrenia.^26, 27^ Given the necessity to establish effective therapeutic interventions against negative symptoms,^3, 4^ efforts to develop and characterize preclinical models with relevance to this symptom domain are urgently needed.^27^ The present study may help in achieving this goal by extending the prenatal PolyI:C administration model to reward-related phenotypes that are reminiscent of key features of the negative symptomatology. Together with other models, the PolyI:C model may also help to address the question of whether reward deficits are primary pathological features of schizophrenia, or whether they emerge as a consequence of pharmacotherapy. Although antipsychotic drugs can affect reward-related functions,^9^ this class of symptoms has also been documented in unmedicated patients with schizophrenia.^14^ Given the known impact of prenatal PolyI:C exposure on early neurodevelopmental processes and subsequent brain maturation,^18, 19^ our study provides preclinical evidence for the hypothesis that at least some reward deficits typically seen in individuals with schizophrenia could arise as a pathological consequence of neurodevelopmental insults during pregnancy, such as those elicited by maternal infection.

Related to this, our findings may highlight a possible etiological association linking maternal infection to substance abuse disorder and/or dual diagnosis schizophrenia. Moreover, they may have implications for the development of reward-related pathologies independently of existing diagnostic classifications. In fact, immune-mediated reward deficits, such as those described in the present study, could represent an intermediary phenotype with relevance to various neuropsychiatric disorders characterized by reward abnormalities, including autism,^79^ bipolar disorder^80^ and schizophrenia.^6^ According to this scenario, the adverse effects of maternal immune activation on reward-related behaviors could represent a point of entry into a disturbed reward system, whereas the specificity of subsequent disease would be further influenced by additional genetic or environmental factors. This hypothesis would be in line with the emerging evidence suggesting that maternal infection has an etiological role in a variety of neuropsychiatric and neurological disorders with seemingly remote pathologies.^81, 82^ Against these backgrounds, our findings may encourage future epidemiological studies to determine whether prenatal infection increases the risk of developing discrete neuropsychopathological features (such as reward-related abnormalities) independently of existing diagnostic classifications. Attempts toward this direction are limited to a few studies showing that serologically documented prenatal infection or inflammation is associated with the presence of discrete neuroanatomical or neurocognitive dysfunctions in patients with schizophrenia.^83, 84^

In conclusion, our study demonstrates abnormal context–reward associations in an immune-mediated neurodevelopmental mouse model relevant to schizophrenia and related disorders. These deficits likely stem from an inability to retrieve previously established context–reward associations and to integrate such information for appropriate goal-directed behavior, but not from anhedonia, neophobia or primary defects in reinforcement learning and/or incentive salience attribution. As currently available pharmacotherapies have thus far failed to improve reward-related deficits in schizophrenia, our epidemiologically informed mouse model may provide an important preclinical platform for the establishment and characterization of novel therapeutic intervention against these symptoms.
