# Supplementary material for: Abnormal context–reward associations in an immune-mediated neurodevelopmental mouse model with relevance to schizophrenia
Source: Transl Psychiatry. 2015 Sep 15;5(9):e637–. doi: 10.1038/tp.2015.129 (PMC5068811; doi:10.1038/tp.2015.129)
Supplement: Supplementary Information [file tp2015129x1.doc]

***Supplementary Information***

**Abnormal Context-Reward Associations in an Immune-Mediated Neurodevelopmental Mouse Model with Relevance to Schizophrenia**

Marie A. Labouesse*, Wolfgang Langhans, Urs Meyer

*Correspondence: Marie A. Labouesse (marie-labouesse@ethz.ch)

ETH Zurich, Schorenstrasse 16, 8603 Schwerzenbach, Switzerland

Tel.: +41 44 655 74 50; Fax.: +41 44 655 72 06.

**Supplementary Results**


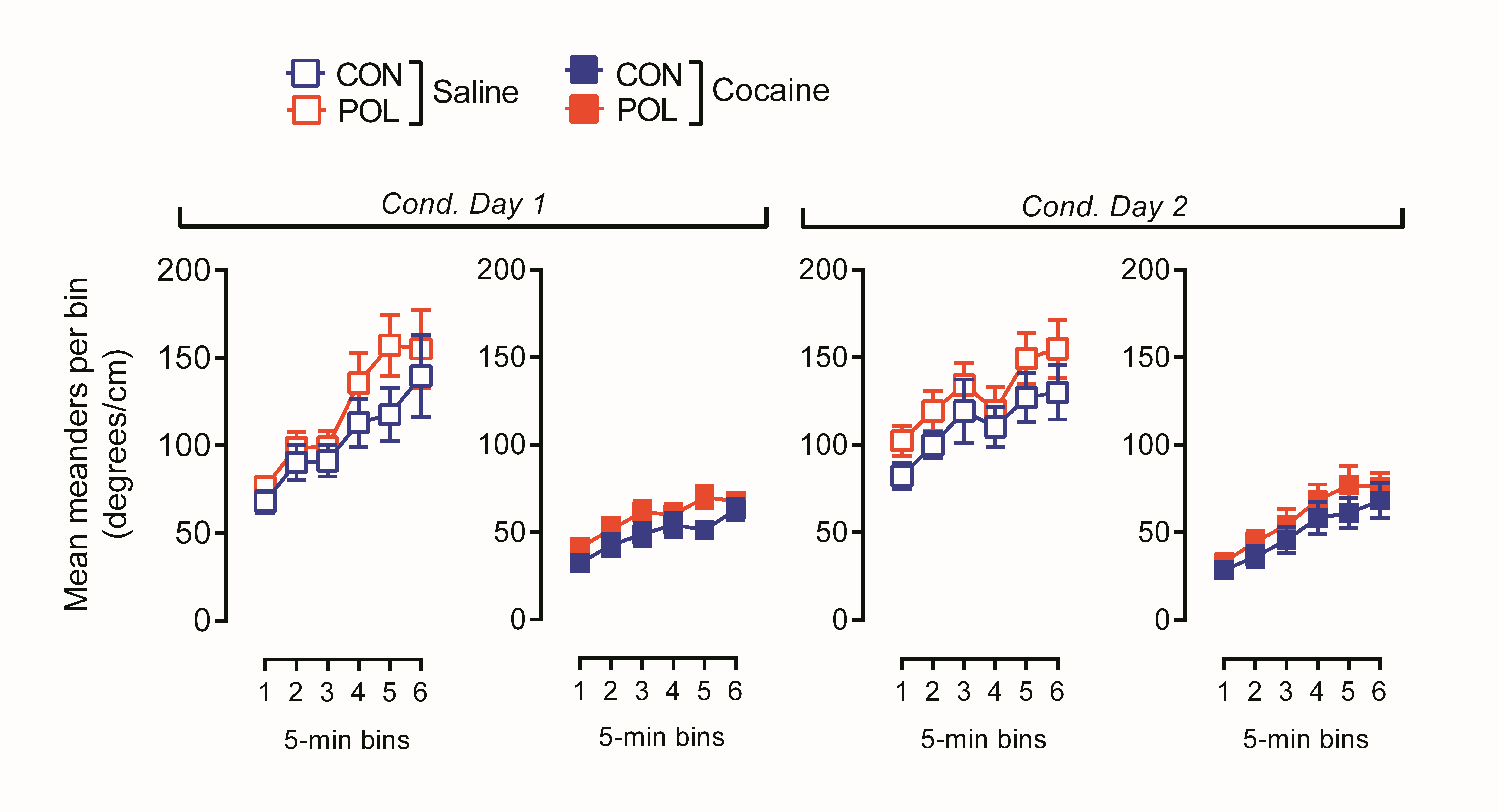


**Figure S1.** Prenatal immune activation does not affect locomotor patterning elicited by cocaine. The line plots show mean meanders (degrees/cm) in the saline-paired and cocaine-paired chambers as a function of 5-min bins on conditioning days 1 and 2. Mean meanders are defined as the degrees of turn angle divided by distance moved. They are a proxy of behavioral patterning and determine the extent to which animals change directions during locomotor activity. Cocaine administration increased locomotor patterning as compared to saline (low meander numbers in cocaine vs. saline: F_(1,34)_ = 87.91, *p* < 0.001), but no differences emerged between offspring born to PolyI:C- or saline-treated mothers. *N*(CON) = 18, *N*(POL) = 18. All values are means±SEM.

--------------------------------------------------------------------------------------------------------------------------------

**Supplementary Discussion**

We have previously shown that exposure to prenatal immune activation in late gestation (gestational day 17; GD17) induces hypersensitivity to the locomotor effects of amphetamine in the adult offspring.^1,2^ The same manipulation on GD17 does not, however, lead to sensorimotor gating deficiencies in the form of reduced prepulse inhibition (PPI) of the acoustic startle reflex,^1^ which is another behavioral readout highly sensitive to dopaminergic imbalances.^3^ Indeed, prenatal poly(I:C)-induced PPI deficits in mice are more robustly induced by immunological challenges taking place earlier during gestation.^1,2^ These findings, together with the data presented in this manuscript (see main text), suggest that prenatal immune activation in late gestation does not simply lead to hyperdopaminergia and associated behavioral impairments. Rather, late prenatal immune activation in mice leads to a complex pattern of neurochemical changes in cortico-striatal circuits in general, and in the accumbal dopamine system in particular.^4^

The attenuation of the locomotor sensitivity to cocaine in offspring born to immune-challenged mothers may, at first glance, seem at odds with our previous findings showing potentiated locomotor reactions of immune-challenged offspring to acute systemic amphetamine treatment.^1,2^ These seemingly paradoxical effects may be best explained by the inherent differences in the molecular mechanisms underlying the effects of cocaine and amphetamine at monoaminergic synapses in general, and at accumbal dopaminergic synapses in particular. While amphetamine can potentiate dopaminergic activity via multiple mechanisms, including increased vesicular release, dopamine reuptake blockade as well as reverse transport of cytoplasmic dopamine via the dopamine transporter (DAT),^5,6^ cocaine exclusively blocks synaptic dopamine reuptake (at least at low concentrations). Hence, unlike amphetamine, cocaine acts to increase synaptic dopamine in a manner that is dependent on vesicular dopamine release induced by neuronal activity.^7^

We have previously shown that prenatal poly(I:C) exposure in late gestation leads to increased levels of striatal tyrosine hydroxylase, the rate-limiting enzyme for dopamine synthesis in the adult offspring.^2^ These findings provide an indirect indication of increased dopamine synthesis and contents in nucleus accumbens dopaminergic neurons. It is thus feasible to assume that amphetamine administration causes more pronounced presynaptic dopamine release in poly(I:C)-exposed mice relative to controls, thereby potentiating the locomotor response to the drug through mechanisms involving altered reverse transport of cytoplasmic dopamine and/or release of vesicular dopamine. In addition, recent unpublished data from our group have identified reductions in accumbal levels of DAT in prenatally immune challenged offspring (Vuillermot and Meyer: unpublished). Because the effects of cocaine are critically dependent on availability of DAT, these findings may provide a potential explanation for the reduced locomotor activity induced by cocaine in these animals. Further studies are needed to examine this hypothesis directly.

**References for the Supplementary Discussion**

1. Meyer U, Nyffeler M, Yee BK, Knuesel I, Feldon J. Adult brain and behavioral pathological markers of prenatal immune challenge during early/middle and late fetal development in mice. *Brain Behav Immun* 2008a; **22**:469-486.
2. Meyer U, Feldon J. Epidemiology-driven neurodevelopmental animal models of schizophrenia. *Prog Neurobiol* 2010; **90**:285-326.
3. Swerdlow NR, Braff DL, Geyer MA. Animal models of deficient sensorimotor gating: what we know, what we think we know, and what we hope to know soon. *Behav Pharmacol* 2000; **11**:185-204.
4. Bitanihirwe BK, Peleg-Raibstein D, Mouttet F, Feldon J, Meyer U. Late prenatal immune activation in mice leads to behavioral and neurochemical abnormalities relevant to the negative symptoms of schizophrenia. *Neuropsychopharmacology* 2010; **35**:2462-2478.
5. Jones SR, Gainetdinov RR, Wightman RM, Caron MG. Mechanisms of amphetamine action revealed in mice lacking the dopamine transporter. *J Neurosci* 1999; **18**:1979-1986.
6. Sieber BA, Kuzmin A, Canals JM, Danielsson A, Paratcha G, Arenas E *et al*. Disruption of EphA/ephrin-a signaling in the nigrostriatal system reduces dopaminergic innervation and dissociates behavioral responses to amphetamine and cocaine. *Mol Cell Neurosci* 2004; **26**:418-28.
7. Heikkila RE, Orlansky H, Cohen G. Studies on the distinction between uptake inhibition and release of (3H)dopamine in rat brain tissue slices. *Biochem Pharmac*ol 1975; **24**:847-852.

……………………………………………………………………………………………………………………………………………………

**Supplementary Methods**

***Animals***

C57BL6/N mice were used throughout the study. Female and male breeders were obtained from Charles River Laboratories (Sulzfeld, Germany) at the age of 10–14 weeks. Breeding began after 2 weeks of acclimatization to the animal holding rooms, which were temperature- and humidity-controlled (21 ± 1°C, 55 ± 5%) facilities under a reversed light–dark cycle. All animals had *ad libitum* access to food (Kliba 3430, Kaiseraugst, Switzerland) and water. All procedures described in the present study had been previously approved by the Cantonal Veterinarian's Office of Zurich. All efforts were made to minimize the number of animals used and their suffering.

***Maternal Immune Activation During Pregnancy.***

For the purpose of the maternal immunological manipulation, C57BL6/N female mice were subjected to a timed mating procedure as described previously.^8-11^ Pregnant dams on gestation day (GD) 17 were randomly assigned to receiving either a single injection of PolyI:C (potassium salt; Sigma-Aldrich, Buchs, St. Gallen, Switzerland) or vehicle. PolyI:C (5 mg/kg) was dissolved in sterile pyrogen-free 0.9% NaCl (= vehicle) solution to yield a final concentration of 1 mg/ml and was administered via the intravenous (i.v.) route at the tail vein under mild physical constraint.

The selected gestational window (i.e., GD 17) in mice corresponds roughly to the middle of the second trimester of human pregnancy, respectively, with respect to developmental biology and percentage of gestation from mice to human.^12^ It was selected based on our previous findings showing that GD17 PolyI:C treatment is capable of inducing neurochemical and behavioral deficits relevant to the negative symptoms, including reduced prefrontal dopamine levels and social interaction impairments.^13^ We have previously verified the effectiveness of this PolyI:C administration protocol in mice in terms of the elicited cytokine-associated inflammatory response in maternal and fetal tissue.^9,10^

***Allocation and Testing of Offspring***

All offspring were weaned and sexed on postnatal day (PND) 21. Littermates of the same sex were caged separately and maintained in groups of 3-5 animals per cage as described above. For each behavioral test, the offspring stemmed from multiple independent litters (minimum *N* = 8 for each prenatal treatment) to avoid possible confounds arising from litter effects. Both male and female offspring were included in all the tests described below in order to assess potential sex-dependent effects of the prenatal immunological manipulation. A *first* cohort of PolyI:C and control offspring was subjected to the conditioned place preference test for sucrose (*N*=35 [17 males, 18 females] in each experimental group). A *second* cohort of PolyI:C and control offspring was used to measure sucrose preference and neophobia, followed by contextual fear conditioning. Half of the animals in cohort 2 was allocated to the sucrose preference test (*N*(control) = 8 [4 males, 4 females], *N*(PolyI:C) = 8 [4 males, 4 females]), and the other half to the sucrose neophobia test (*N*(control) = 8 [4 males, 4 females], *N*(PolyI:C) = 8 [4 males, 4 females]), in order to avoid multiple sucrose exposures that might affect neophobic responses. Following a resting period of 1 week, all animals from cohort 2 were then subjected to the contextual fear conditioning test (*N*(control) = 16 [8 males, 8 females], *N*(PolyI:C) = 16 [8 males, 8 females]). A *third* cohort of PolyI:C and control offspring (*N*=18 [8 males, 10 females] in each experimental group) was used for the assessment of conditioned place preference for cocaine. Estimations of sample size were based on previous studies from our laboratory,^8-10,13,14^ except for the conditioned place preference experiments where the sample size was based on published studies from other research groups^15,16^ and our own preliminary analyses in normal wild-type animals (unpublished data). All behavioral testing commenced when the offspring reached the adult stage of development (i.e., PND 80 onwards till PND 120) and occurred in a randomized and counterbalanced fashion so that equal numbers of control and PolyI:C offspring were tested in each testing squads and across the entire duration of testing.

***Conditioned Place Preference for Sucrose***

Conditioned place preference (CPP) is a well-established test to assess the integrity of reward function in rodent models.^17,18^ In particular, this test detects the ability to form context-reward associations, which in turn are dependent on various behavioral processes such as reinforcement learning, attribution of incentive salience for conditioned cues and rewards, as well as translation of relevant information into goal-directed behaviors. In a first series of investigations, we used sucrose as the primary source of reinforcement. Sucrose is considered a natural reinforcer, i.e. it belongs to the category of reinforcers that are physiologically required for the survival of the organism such as food, water, sex and nurturing.^19^

The experimental procedure used for the sucrose CPP was adapted from Perello et al.^16^ A two-chamber apparatus was used, which consisted of two dimly-lit conditioning chambers (25 cm (length) × 16 cm (width) × 16 cm (height)) that differed from each other in wall patterns (large vertical black and white stripes vs. large black filled circles on a white background) and floor textures (thin grid metal mesh vs. metal floor with small 2-mm spaced holes). Two white round glass plates were placed in the center of each chamber, where 30% sucrose (Sigma-Aldrich, Buchs, Switzerland) or regular tap water was poured freshly prior to each session. A plastic transparent lid that covered both chambers prevented the animals from escaping. A digital camera was mounted above the apparatus and images were transmitted at a rate of 5 Hz to a personal computer running the Ethovision (Noldus IT, Wageningen, The Netherlands) software allowing the tracking of the animal’s position. The output of the camera was also transmitted to the WINTV-2000 program for video recording. Three days prior to beginning the CPP test, mice were exposed to the 30% sucrose solution to avoid neophobia by giving them *ad libitum* access to a sucrose bottle in addition to their regular drinking water bottle. Prior to each test session, mice were mildly food-deprived (but not water-deprived) for 2 hours in order to baseline hunger levels in all mice.

Two pre-conditioning sessions were performed on day 1 (one in the morning and one in the afternoon), during which a single mouse was randomly placed in one of the two chambers of the CPP apparatus and allowed free access to both chambers for 25 min in the absence of sucrose or water. This procedure allowed the animals to habituate to the novelty of the new environment and to the bi-daily food deprivation and experimental schedules. Exploratory time in the two chambers was evaluated to determine whether each individual mouse displayed an intrinsic preference for one of the two chambers. The assignment of conditioning chambers to either sucrose or water was performed in an unbiased manner across treatment groups, i.e. in a way that in each treatment group half of the animals were conditioned to their initially preferred chamber, while the other half was conditioned to their initially non-preferred chamber. This ensured that, for each prenatal treatment group, the average percent time spent in each of the two chambers were comparable (approximately 50.0%, data not shown). Animals that did not enter both chambers on the pre-conditioning day were excluded from any further analyses.

Conditioning sessions were performed on days 2 to 5 with two sessions per day. All animals were allowed a 4-hour resting period between morning and afternoon sessions. In the morning sessions, mice were confined to one conditioning chamber in the presence of sucrose or water for 20 min, while they were confined to the other chamber in the afternoon sessions. To prevent animals from developing associations between the rewards (sucrose S vs. water W) and the time of day (morning vs. afternoon), the exposure to the different rewards were modified in a counterbalanced fashion across days, yielding four different orders of exposure:

- *1)* Day 1 (S morning/W afternoon), Day 2 (W/S), Day 3 (W/S), Day 4 (S/W)
- *2)* Day 1 (S/W), Day 2 (W/S), Day 3 (S/W), Day 4 (W/S)
- *3)* Day 1 (W/S), Day 2 (S/W), Day 3 (S/W), Day 4 (W/S)
- *4)* Day 1 (W/S), Day 2 (S/W), Day 3 (W/S), Day 4 (S/W)

These orders of exposure were counterbalanced across prenatal treatment groups and across sucrose-paired chamber assignments.

A final test session was performed on day 6, during which the mice were initially placed in the chamber paired with water and then allowed free access to both conditioning chambers for 25 min, in the absence of sucrose or water. Exploratory time in the two chambers was evaluated so as to determine whether a preference towards the sucrose-paired chamber would emerge as a result of conditioning. The investigator was blind to the prenatal experimental treatment group during the entire duration of testing.

In addition, a number of other variables were measured in order to determine the existence of possible confounders in the assessment of CPP performance. Total locomotor activity was determined during the test session to ensure that differences in place preference did not emerge secondary to differences in locomotor activity. Two animals were excluded based on a Grubbs outlier test for locomotor activity in the test session. Locomotor activity across conditioning sessions was also measured to determine whether differences in these measures would emerge as a result of prenatal treatment. To assess the successful acquisition of context-reward associations at the end of conditioning sessions, a “conditioning score” was determined by measuring the latency to eat sucrose (vs. water) in the first 5 minutes of the last conditioning session. Such analyses were conducted by an experimenter blind to the experimental treatment and scored manually by the analysis of videotapes. If an animal did not consume the sucrose (or water) within the 5 first minutes of the test, it was given a score of 300 sec (5 min). Finally, food and fluid intake were measured in the same cohort of animals (at the end of the CPP procedure) using the same food deprivation paradigm (2 hours food deprivation) to determine whether prenatal PolyI:C would modulate hunger or thirst states and thus possibly confound the results of the CPP test.

***Sucrose Preference Test***

Because the development of place preferences can emerge as a result of abnormal anhedonic behavior we assessed the hedonic response to sucrose using a standard sucrose preference test adapted from previous studies.^13,20^ The sucrose preference test is based on the observation that rodents typically show a preference for a sweet sucrose solution when presented with a free choice between the sucrose solution and water. A reduction in this preference is commonly considered as an indication of anhedonia. Animals were provided with two polypropylene 15mL drinking tubes in their home cages, and they were moved from grouped to single housing 3 days before commencement of the test (habituation phase) and throughout the testing period. They were first familiarized with drinking water from the two tubes during which water consumption was measured every 24 h for 3 days (habituation phase). The sucrose preference test then begun on the next day and lasted for 24 h. During the test, the mice were allowed free constant access to liquid from the two drinking tubes and food located on the cage top. One drinking tube was filled with a 30% sucrose solution and the other with water. The relative left-right positions of the sucrose and water tubes were counterbalanced within each group. Sucrose preference was indexed by a percentage score [sucrose consumption / (total liquid consumption) × 100%]. In addition, we also recorded and analyzed the total fluid consumption in order to compare general fluid intake between the experimental groups. The investigator was blind to the prenatal experimental treatment group during the entire duration of testing.

***Sucrose Neophobia Test***

The food neophobia test is based on the observation that mice, like most other rodents, typically avoid consuming novel foods/solutions upon first presentation.^21^ Mice with high levels of innate anxiety typically take longer to begin consuming an unfamiliar food/solution compared to less anxious mice.^21^ Anxiety-related sucrose neophobia could represent a major confounder in the analysis and interpretation of the CPP test for sucrose. Although we made every effort to avoid such effects by pre-exposing the animals to the sucrose solution prior to CPP testing (see above), we further evaluated this possibility by directly measuring sucrose neophobia.

The apparatus consisted of a rectangular arena (42 cm long, 26 cm wide) surrounded by transparent walls (15 cm high). The apparatus was placed in a testing room under bright light (50 lux). The test was performed on 2 consecutive days: On each day, subjects were food-deprived for 2 h prior to placing them into the test apparatus. On the first day, the animals were placed into the test arena and allowed to freely explore it for 15 min. This served to habituate the animals to the test apparatus and reduce potential confounds resulting from increased locomotor responses to novelty. The actual food neophobia test was then performed on the next day, during which the novel food (30% sucrose solution poured in a glass plate) was presented in the test arena for 10 min. If an animal started consuming and continued for 3 s, the trial was stopped and the animal was brought back into the colony room. If an animal did not start eating within the 10-min period, the trial was interrupted and the latency to eat was scored as 10 min. The latency to consume the sucrose solution was scored by an experimenter blind to the treatment group. The investigator was blind to the prenatal experimental treatment group during the entire duration of testing.

***Contextual Fear Conditioning***

Based on our initial observations that prenatal immune activation disrupts positive contextual conditioning, we further performed a negative contextual conditioning task in the form of context-fear associative learning.

The apparatus comprised 4 Coulbourn Instruments as fully described elsewhere.^22^ The algorithm of the freezing response detection procedure was validated previously and is fully described elsewhere.^23^ The contextual fear test was adapted from protocols established by Deacon et al.^24^ and consisted of two phases, separated by 24 h: On the first day, the animals were placed into the designated chambers and received 3 electric foot shocks (1-s, 0.3 mA) separated by 30-s intervals. The first shock was delivered after an initial 3-min habituation period, during which no stimulus other than the house light was presented. The animals were removed from the conditioning chambers and brought back to their home cages 30 s after the last foot shock. During the conditioning day, the amount of freezing during each 30-s post-shock period provided a measure for the evaluation of the acquisition of conditioning. On the second day, the animals were placed back into the same conditioning chambers for a period of 6 min, during which their freezing behavior was recorded. This served as a test of measuring conditioned fear towards the context. The expression of context freezing during the 6-min test period was indexed as percent time freezing and expressed as a function of 30-s bins. The investigator was blind to the prenatal experimental treatment group during the entire duration of testing.

***Conditioned Place Preference for Cocaine***

To test whether the effects of prenatal immune activation would extend to drug reinforcers, we subjected prenatal PolyI:C and control offspring to a CPP experimental task using the same two-chamber apparatus as described above, whereby the sucrose reinforcer was replaced by a drug reinforcer in the form of cocaine administration**.** The experimental procedure was adapted from Paris et al.^15^ A digital camera was mounted above the apparatus and images were transmitted at a rate of 5 Hz to a personal computer running the Ethovision (Noldus IT, Wageningen, The Netherlands) software allowing the tracking of the animal’s position.

A pre-conditioning session was performed on day 1, during which a single mouse was administered a saline i.p. injection and then randomly placed in one of the two chambers of the CPP apparatus and allowed free access to both chambers for 20 min. This procedure allowed the mice to habituate to the novelty of the new environment and to the bi-daily injection and experimental schedules. Exploratory time in the two chambers was evaluated to determine whether each individual mouse displayed an intrinsic preference for one of the two chambers. The assignment of conditioning chambers to either saline or cocaine was then performed in an unbiased manner across treatment groups, i.e. in a way that in each treatment group approximately half of the animals were conditioned to their initially preferred chamber, while the other half were conditioned to their initially non-preferred chamber. Such assignment protocol is particularly important for CPP experimental paradigms, because a biased protocol (i.e. all animals are assigned their non-preferred chamber for cocaine-pairing) can lead to bias in interpretation.^25^ Because the animals assigned to the cocaine CPP test displayed an overall intrinsic preference for one of the two chambers (the “*Stripes*” chamber), a common phenomenon described by many others,^18,25^ it was not possible to obtain perfectly equivalent percent exploratory times (50/50%) between cocaine- and saline-paired chambers using the unbiased chamber assignment described above, i.e. there was a (slight) bias towards one of the two chambers (values of 40% rather than the expected 50%, data not shown). This marginal bias emerged, however, independently of the prenatal history. Animals that did not enter both chambers were excluded from any further analyses.

Conditioning sessions were performed on days 2 and 3 with two sessions per day. All animals were allowed a 4-hour resting period between morning and afternoon sessions. In the morning sessions, mice were i.p administered with either cocaine (10mg/kg) or saline and confined to one conditioning chamber for 30 min, while they were confined to the other chamber in the afternoon sessions. In order to allow a minimum of 24-h between the two cocaine sessions, animals were either assigned to receiving cocaine during morning sessions and saline during afternoon sessions, or the opposite. Such exposure patterns were counterbalanced across treatment groups and across cocaine-paired chamber assignments. The investigator was blind to the prenatal experimental treatment group during the entire duration of testing.

A final test session was performed on day 4 at mid-day, during which mice were i.p. administered an innocuous injection in the form of saline. Animals were then immediately placed in the chamber paired with saline and then allowed free access to both conditioning chambers for 20 min. Exploratory time in the two chambers was evaluated so as to determine whether a preference towards the cocaine-paired chamber would emerge as a result of conditioning.

Total locomotor activity was determined during the test session to ensure that differences in place preference did not emerge secondary to differences in locomotor activity. Locomotor activity across conditioning sessions was also measured to assess whether the prenatal treatment may affect the locomotor reactions to cocaine during the conditioning sessions, which in turn could represent a possible confounder for the analysis and interpretation of the CPP results. Behavioral patterning, as a proxy for stereotypical horizontal locomotor responses to cocaine, was also assessed during the conditioning sessions. Low meandering (mean meanders per bin in degrees/cm) is a sign of high behavioral patterning.^26^

***Statistical analyses***

All statistical analyses were conducted using the statistical software StatView (version 5.0) or SPSS (version 22) implemented on a PC running the Windows XP operating system. Statistical significance was set at *p* < 0.05. All data were presented as means±SEM. All data met the assumptions of normal distribution and equality of variance, or were transformed to meet these criteria. All data were analyzed using Student’s *t-*tests (two-tailed), parametric analysis of variance (ANOVA) or analysis of covariance (ANCOVA), followed by Fisher's least significant difference (LSD) post-hoc group comparisons/restricted ANOVA whenever appropriate, except for one exception where transformation did not yield normal distribution so that a non-parametric Mann-Whitney test was used. Preliminary analyses revealed no significant sex × treatment interactions in all the dependent measures so that the two sexes were collapsed in the final presentation of the data to enhance statistical power. All experiments presented in this manuscript have been replicated at least once in previous studies (Meyer et al., 2005, 2006a,b, 2009 and unpublished data), except for the conditioned place preference experiments where a high number of experimental animals were purposely included.

In the analyses of the sucrose CPP, the time spent in the sucrose vs. the time spent in the water-paired chambers on the test day was analyzed using a 2 × 2 × 5 (prenatal treatment × chamber × bins). The relative difference in the exploratory time between the sucrose- and the water-paired chamber was analyzed using a non-parametric Mann-Whitney test. Total locomotor activity in the test session was log-transformed and analyzed using a 2 × 5 (prenatal treatment × bins) repeated-measures ANOVA. Locomotor activity during conditioning sessions was analyzed using a 2 × 2 × 4 (prenatal treatment × chamber × days). The latency to consume sucrose on the last conditioning day was log-transformed and analyzed using a Student’s *t*-test. In the sucrose preference test, sucrose preference and total fluid intake were assessed using Student’s *t*-tests with prenatal treatment as the independent variable. In the food neophobia test, the latency to consume sucrose was analyzed using a Student’s *t*-test with prenatal treatment as the independent variable. In the analyses of the cocaine CPP, the time spent in the cocaine-paired chamber vs. the time spent in the saline-paired chambers on the test day were analyzed using a 2 × 2 × 4 (prenatal treatment × chamber × bins) repeated-measures ANOVA. The relative difference in the exploratory time between the cocaine-paired vs. saline-paired chamber was analyzed using a one-way ANOVA. Total locomotor activity in the test session was analyzed using a 2 × 4 (prenatal treatment × bins) repeated-measures ANOVA. Locomotor responses to cocaine or saline during the conditioning sessions were square-root transformed and analyzed using a 2 × 2 × 6 × 2 (prenatal treatment × drug × bins × conditioning days) repeated-measures ANOVA. Mean meanders during the conditioning sessions were analyzed using a 2 × 2 × 6 × 2 (prenatal treatment × drug × bins × conditioning days) repeated-measures ANOVA.

**References for Supplementary Methods**

1. Meyer U, Feldon J, Schedlowski M, Yee BK. Towards an immuno-precipitated neurodevelopmental animal model of schizophrenia. *Neurosci Biobehav Rev* 2005; **29**: 913-947.
2. Meyer U, Feldon J, Schedlowski M, Yee BK. Immunological stress at the maternal-foetal interface: a link between neurodevelopment and adult psychopathology. *Brain Behav Immun* 2006a; **20**:378-388.
3. Meyer U, Nyffeler M, Engler A, Urwyler A, Schedlowski M, Knuesel I *et al.* The time of prenatal immune challenge determines the specificity of inflammation-mediated brain and behavioral pathology. *J Neurosci* 2006b; **26**:4752-4762.
4. Meyer U, Feldon J, Fatemi SH. In-vivo rodent models for the experimental investigation of prenatal immune activation effects in neurodevelopmental brain disorders. *Neurosci Biobehav Rev* 2009; **33**:1061-79.
5. Clancy B, Finlay BL, Darlington RB, Anand KJ. Extrapolating brain development from experimental species to humans. *Neurotoxicology* 2007; **28**:931-937.
6. Bitanihirwe BK, Peleg-Raibstein D, Mouttet F, Feldon J, Meyer U. Late prenatal immune activation in mice leads to behavioral and neurochemical abnormalities relevant to the negative symptoms of schizophrenia. *Neuropsychopharmacology* 2010; **35**:2462-78.
7. Bitanihirwe BK, Weber L, Feldon J, Meyer U. Cognitive impairment following prenatal immune challenge in mice correlates with prefrontal cortical AKT1 deficiency. *Int J Neuropsychopharmacol* 2010a; **13**:981-96.
8. Paris JJ, Carey AN, Shay CF, Gomes SM, He JJ, McLaughlin JP. Effects of conditional central expression of HIV-1 tat protein to potentiate cocaine-mediated psychostimulation and reward among male mice. *Neuropsychopharmacology* 2014; **39**:380-8.
9. Perello M, Sakata I, Birnbaum S, Chuang JC, Osborne-Lawrence S, Rovinsky SA *et al.* Ghrelin increases the rewarding value of high-fat diet in an orexin-dependent manner. *Biol Psychiatry* 2010; **67**:880-6.
10. Huston JP, Silva MA, Topic B, Müller CP. What's conditioned in conditioned place preference? *Trends Pharmacol Sci* 2013; **34**:162-6.
11. Tzschentke TM. Measuring reward with the conditioned place preference (CPP) paradigm: update of the last decade. *Addict Biol* 2007; **12**:227-462.
12. Olsen CM. Natural rewards, neuroplasticity, and non-drug addictions. *Neuropharmacology* 2011; **61**:1109-22.
13. Slattery DA, Markou A, Cryan JF. Evaluation of reward processes in an animal model of depression. *Psychopharmacology (Berl)* 2007; **190**:555-68.
14. File SE. Factors controlling measures of anxiety and responses to novelty in the mouse. *Behav Brain Res* 2001; **125**:151-7.
15. Schwendener S, Meyer U, Feldon J. Deficient maternal care resulting from immunological stress during pregnancy is associated with a sex-dependent enhancement of conditioned fear in the offspring. *J Neurodev Disord* 2009; **1**:15-32.
16. Richmond MA, Murphy CA, Pouzet B, Schmid P, Rawlins JN, Feldon J. A computer controlled analysis of freezing behaviour. *J Neurosci Methods* 1998; **86**:91-99.
17. Deacon RM, Bannerman DM, Kirby BP, Croucher A, Rawlins JN. Effects of cytotoxic hippocampal lesions in mice on a cognitive test battery. *Behav Brain Res* 2002; **133**:57–68.
18. Napier TC, Herrold AA, de Wit H. Using conditioned place preference to identify relapse prevention medications. *Neurosci Biobehav Rev* 2013; **37**:2081-6.
19. Fox MA, Panessiti MG, Hall FS, Uhl GR, Murphy DL. An evaluation of the serotonin system and perseverative, compulsive, stereotypical, and hyperactive behaviors in dopamine transporter (DAT) knockout mice. *Psychopharmacology (Berl)* 2013; **227**:685-95.
